# *Salvadora persica*: Nature’s Gift for Periodontal Health

**DOI:** 10.3390/antiox10050712

**Published:** 2021-04-30

**Authors:** Mohamed Mekhemar, Mathias Geib, Manoj Kumar, Radha, Yasmine Hassan, Christof Dörfer

**Affiliations:** 1 Clinic for Conservative Dentistry and Periodontology, School of Dental Medicine, Christian-Albrecht’s University, 24105 Kiel, Germany; stu219512@mail.uni-kiel.de (Y.H.); doerfer@konspar.uni-kiel.de (C.D.); 2 Dr. Geib Private Dental Clinic, Frankfurter Landstraße 79, 61352 Bad Homburg, Germany; geib@drgeib.de; 3 Chemical and Biochemical Processing Division, ICAR—Central Institute for Research on Cotton Technology, Mumbai 400019, India; manoj.kumar13@icar.gov.in; 4 School of Biological and Environmental Sciences, Shoolini University of Biotechnology and Management Sciences, Solan 173229, India; radhuchauhan7002@gmail.com

**Keywords:** *Salvadora persica*, periodontal disease, periodontitis, anti-inflammatory agents, antioxidants, adjunctive periodontal therapy, herbal medicine, oral health

## Abstract

*Salvadora persica* (SP) extract, displays very valuable biotherapeutic capacities such as antimicrobial, antioxidant, antiparasitic and anti-inflammatory effects. Numerous investigations have studied the pharmacologic actions of SP in oral disease therapies but its promising outcomes in periodontal health and treatment are not yet entirely described. The current study has been planned to analyze the reported effects of SP as a support to periodontal therapy to indorse regeneration and healing. In consort with clinical trials, in vitro investigations show the advantageous outcomes of SP adjunctive to periodontal treatment. Yet, comprehensive supplementary preclinical and clinical investigations at molecular and cellular levels are indispensable to reveal the exact therapeutic mechanisms of SP and its elements for periodontal health and therapy.

## 1. Introduction

Over the last few years, global awareness on the application of herbal therapies to treat diverse conditions has been rising expansively owing to their encouraging results and scarce adverse effects [1]. As clarified by the World Health Organization (WHO), the majority of the global general population, mostly in emerging countries and societies, hinge on natural medicine and traditional herbal therapies for their focal treatment and health care of various conditions. Consequently, the WHO has inspired developing nations to embrace therapeutic herbs as an additional resource to upsurge the success of health care systems [1,2]. Amongst the evidence-based plant therapies, positively graded as a “miracle twig” is *Salvadora persica* (SP) [3].

SP, frequently known also as Miswak, is a member of the plant family *Salvadoracea* [4]. It is mainly distributed in dry and subtropical regions of Africa and the Middle East, as well as the Indian subcontinent [5]. The fresh leaves, twigs and roots of its small tree can be added to the daily diet and are applied in traditional herbal therapy for asthma, scurvy, cough, rheumatic illnesses, oral hygiene and other conditions [4,6]. The use of SP originates in the Pre-Islamic and Islamic era, as it was introduced by Arabic and Islamic societies as a predecessor of toothbrushes to clean the teeth and promote a good oral hygiene [7]. The advantageous properties of SP regarding dental and oral health can be explained by its mechanical action if used for brushing in addition to its pharmacologic active constituents. These encompass chemically active substances as tannins that inhibit glucosyltransferase enzyme to diminish plaque and periodontal diseases, and resins that protect against dental caries [8] Moreover, the antimicrobial, anti-inflammatory and antioxidant activities of SP have been ascribed to several detectable substances in its natural extracts such as potassium and sodium chloride, as well as Salvadorine, vitamin C, Salvadourea, silica, Saponins and different minerals [8,9].

Diseases of the periodontal structures have besieged humans worldwide since early history. Paleo-pathological studies have explained that periodontal induced bone loss has impacted early populations in different regions worldwide [10]. Gingivitis is considered the first stage of periodontal disease and inflammation and initiates in the gingiva due to accumulated plaque and microorganisms between the gingival line and teeth. This responsive disease is revocable through an improved oral hygiene. In periodontitis the illness further progresses into an irreversible and chronic inflammatory condition finally leading to bone resorption. During this pathophysiological multifactorial process the host immune response and the invading bacteria provoke the periodontal destruction via losing bony attachments, subsequently promoting loss of alveolar bone at the affected sites [11]. Non-surgical periodontal treatment can show therapeutic effects in mild to moderate periodontal disease, whereas surgical periodontal therapy is needed in progressive disease conditions [10]. Supplementary to mechanical debridement in periodontal treatment, various drug delivery methods, mouth rinses, irrigating solutions, and long-term drug release systems are commonly used for therapeutic agent administration [10,12]. Furthermore, routine periodontal therapy combined with an adjunctive therapeutic mediator has been shown to greatly improve patient outcomes as compared to mechanical debridement only [10,13]. Modern chemotherapeutic agents showed impressive efficacy in the treatment of periodontal disease, but they also had a variety of intolerable side effects, including strong taste changes, tongue and tooth discoloration, unwanted antibacterial resistance, and higher product prices [14,15].

As a result, the use of natural and herbal products as SP for periodontal therapy has received much interest lately, and could present many profits, particularly for people from lower socioeconomic backgrounds all over the world [16,17].

In order to explore the subject of SP application in the course of periodontal treatment and its mode of action, the search terms *Salvadora persica*, Meswak, Miswak, *Salvadora Indica*, Arak, or Pilu combined with periodontal therapy, treatment, disease and regeneration, or periodontitis were entered into PubMed, Google Scholar, MEDLINE and the Cochrane database. The evidence-based medicine hierarchical structure was then applied to the study. The review identified and reported laboratory-based experiments, in vivo studies, and clinical trials. Because this study was not designed to be a systematic review, no specific final recommendations were made. As more investigations are required presently in this field, this article attempts to utilize the best available evidence to reveal the scientific position of the subject and the necessity for more investigations to intensify the current understanding.

## 2. Periodontitis and Periodontal Treatment

### 2.1. Periodontitis as a Worldwide Health Burden

Periodontitis is a progressive destructive condition of the Periodontium, which holds the tooth-supporting structures inside the oral cavity such as alveolar bone, root cementum, and periodontal ligament fibers. The loss of clinical periodontal attachments and radiographically diminished alveolar bone with clinically visible periodontal pockets, gingival bleeding, and inflammation are its main destructive effects. If periodontitis is left untreated, it can lead to tooth loss; however, this can be avoided with a correct treatment plan [18]. Periodontal disease is a global prevalent burden on general wellbeing and health-related quality of life, since it can trigger tooth loss, which can contribute to masticatory and verbal incapacitation, as well as esthetic and psychological complications. Furthermore, edentulism in association with periodontal disease accounts for major dental rehabilitation costs, indicating a serious financial problem that affects a significant number of people around the world [19]. As a chronic inflammatory and multifactorial disorder related to dysbiosis of dental plaque biofilm, periodontal inflammation is considered the most common chronic inflammatory disorder of all human societies. As previously stated, the global age-standardized prevalence of severe periodontal disease in the last 10 years was nearly 11%, indicating that periodontitis is one of the most common diseases worldwide [20], while other studies have shown that the global prevalence of mild periodontal disease is as high as 50% [21]. If periodontitis is left untreated or is not properly handled medically, it has the potential to cause more years of debility and malfunction than all other human diseases [22]. Furthermore, periodontal diseases are linked to a variety of systemic diseases that can result in death and a variety of serious health problems, including cardiovascular diseases [23], diabetic complications [24] and various adverse consequences during pregnancy [25]. Periodontal disorder has been estimated to cost more than $50 billion globally, with an additional $25 billion spent on direct and indirect treatment costs [22]. Periodontitis-related edentulism contributes significantly to the economic burden of dental problems due to the rehabilitation treatments required following tooth loss, resulting in significant rises in the overall economic burden associated with dentistry. The total cost of dental care was recently estimated to be $544.41 billion, with $356.80 billion in direct costs and $187.61 billion in indirect costs [26].

### 2.2. Strategies for Periodontal Therapy

#### 2.2.1. Mechanical Therapy

Dental plaque includes a combination of food deposits, bacteria and minerals. Since plaque can harden into dental calculus or linger in hard-to-reach areas of the dentition, both forms of deposits can be difficult to remove with toothbrushing or flossing, causing inflammation of the gingival tissues as a first stage that leads to periodontal disease [11]. Periodontal scaling is the most efficient method for halting this damaging inflammatory process. Dentists use dental scaling techniques to remove soft and hard dental residues, as well as stains, from the affected teeth’s root and crown surfaces [27]. Scaling and root planning has become the gold standard in nonsurgical periodontitis mechanical therapy. Many clinical studies have shown that it significantly decreases the number of microorganisms in periodontal pockets and enhances clinical outcomes such as clinical attachment, bleeding on probing, and probing depths [28]. Scaling and root planning can be performed with a variety of dental devices, including manual scalers and curettes, as well as sonic and ultrasonic instruments that help reach deeper into pocket depths and into dental root furcations [27]. Since pathogens may exist inside tissues or deep pockets that are not reachable by instruments, it is not always possible to remove all pathogens during localized scaling and root planning procedures. This lead to the use of antimicrobial and chemotherapeutic agents as adjunctive and complementary therapies in addition to the main mechanical debridement to restore the physiological microbiological profile, control periodontal inflammation, and initiate tissue regeneration [29,30]. Some progressive periodontal treatments, such as laser therapy, are still being investigated. Periodontal laser therapy can be used as an alternative to traditional mechanical periodontal debridement because of its ability to excise, evaporate, and sterilize periodontal pockets. However, more research is required to look into the microbiological recolonization following to laser periodontal treatments [27,31,32].

#### 2.2.2. Chemotherapeutic Periodontal Therapy

##### Host Modulation Therapy (HMT)

Periodontal tissue destruction is initiated by bacterial toxins and plaque, in addition to the important aspect of the host immune response to infections, according to previous histological and clinical findings of periodontitis [33]. This primary immune component in periodontal disease pathogenesis prompted the development of host modulatory therapy (HMT) to modify the host immune response and reduce immune-facilitated damage levels [34].

Several host modulatory agents are offered for chemotherapeutic periodontal treatment, adjunctive to the mechanical therapy by localized or systemic administration, such as doxycycline at sub-antimicrobial dose [35], anti-inflammatory compounds, as steroids, non-steroidal anti-inflammatory drugs (NSAID), anti-TNF and anti-IL1 [36], several growth factors, bisphosphonates, and derivatives of enamel matrix [37,38]. These chemotherapeutic agents all have the ability to mediate the host-immune reaction through various mechanisms, eventually blocking the destructive aspects of inflammation [34]. These mechanisms include anti-inflammatory mediators inhibiting prostaglandins or pro-inflammatory cytokines, tetracyclines and doxycycline inhibiting collagenase, and bisphosphonates inhibiting osteoclast cell activity [27].

##### Antimicrobial Therapy

Due to the fact that mechanical debridement of periodontal tissues and pockets does not permanently destroy all pathogens, lingering pathogens in the periodontal system often recolonize the tissues several weeks after treatment [39]. As a result, in addition to mechanical and surgical therapy, adjunctive application of systemic chemotherapeutic antimicrobial agents has proven to be more effective in the wide-ranging extermination of pathogenic bacteria [40]. Despite the fact that systemic antimicrobial therapy has many encouraging effects in periodontal treatment [41,42], it is usually reserved for patients with rapidly progressing or refractory periodontitis due to its major concurrent disadvantages [27]. The antibiotic’s unpredictable concentration at the intended site, the risk of a rapid decrease in plasma antibiotic concentration below the appropriate therapeutic index, and the development of antibiotic resistance by microorganisms are only a few of these inadequacies [27,42]. Besides that, administering high doses of systemic antibiotics to a large number of patients may cause a variety of side effects [27,42]. Therefore, the production and investigation of localized intra-pocket drug delivery systems for periodontal therapy was endorsed by the aforementioned possible drawbacks of systemically administered antimicrobial chemotherapeutics [30]. This method of localized drug distribution within periodontal pockets resulted in less drug-related side effects, higher drug doses at the target site over longer periods of time, and higher patient compliance [27]. Local drug distribution for antibacterial agents and other forms of chemotherapeutic mediators in periodontal therapy can be achieved by the local application of topical oral gels and solutions, or by the introduction of special delivery systems into periodontal pockets, such as periodontal chips, for a sustained release of the necessary medication concentrations [27,30,40].

##### Herbal Agents for Periodontal Therapy

Herbal remedies and compounds are medicinal plant elements that have been found to have therapeutic benefits both historically and scientifically [10]. There has been an increase in global interest in the use of natural herbs in a number of clinical procedures due to their positive outcomes and lower side effects as compared to systemic medications and other chemotherapeutic agents. [10]. In response to this focus on herbal remedies as well as a broad range of treatments, periodontal treatments have recently introduced the use of various herbal chemotherapeutic agents as an adjunct to scaling and root planning [10,43]. This intended to evade the various adverse effects of modern chemical agents, including the mentioned adverse effects of antibiotics, besides tooth and tongue discolorations [44], taste changes [44] and the high economic burden produced by the drug costs [10]. The herbal and natural agents frequently used for periodontal treatment include *Cinnamomum zeylanicum, Acacia catechu*, *Propolis*, *Mikania glomerate, Mikania laevigata*, *Glycyrrhiza glabra*, *Droserapeltata, Aloe vera*, *Allium sativum*, *Helichrysumitalicum*, *Piper cubeba, Coptidis rhizome*, *Azadirachta indica*, *Nigella Sativa*, *Syzygium aromaticum* and tea tree oil [1,10,31]. Amongst the most promising herbal medications with conspicuous budding future profits for periodontal health and treatment is SP, as a widely used plant in traditional medicine in Africa, the Middle East and Asia for the treatment of a wide range of diseases and conditions [45,46].

## 3. *Salvadora persica* as a Therapeutic Agent

### 3.1. Historical and Cultural Importance of Salvadora persica

SP is a well-established cultural and religious-based herbal therapy for a variety of health conditions that has been identified in various parts of the world [47,48]. SP, a plant native to dry areas of Africa and the Middle East, as well as being widely cultivated in regions across the Indian Peninsula, became a common herbal medicine during various historical periods [7].

The precise origin of mechanical tooth brushing tools is uncertain. However, before the advent of the modern toothbrush, civilized nations used various kinds of brushing devices to clean and protect their teeth. The toothpick and twig brush were among the first instruments invented. [7,49]. Toothpicks have been used since ancient times. They were discovered alongside other toiletry products in Ur, an ancient Babylonian city that flourished around 3500 BC. The Greek sophist Alciphron advocated using a toothpick to clean the “fibrous debris” that stood between the teeth during meals in the second century BC [7]. The Babylonian fiber brush, also known as the chewing stick, was used as early as 3500 BC and may have been the historical forerunner of the modern toothbrush. It was a wooden stick about 5 or 6 inches long. One end was macerated to separate the fibers to produce a brush-like end. With approximately 182 plant species potentially providing chewing sticks, SP was traditionally the most widely used one for this activity [46]. Prior to the arrival of Islam, Arabs extracted chewing sticks from the root of the SP tree and used them as a herbal therapy for oral and dental hygiene [7]. Later, during the early Islamic period, the use of Miswak became part of a refined way of life. Its use as a simple oral hygiene method became absorbed into Islamic religious practice and was a constant Prophetic practice in all daily activities. Since then, the Miswak has played an important role in Islamic hygienic jurisprudence, with Muslim scholars emphasizing its importance in many Prophetic narratives [7]. As a result, Islam had a major influence on the spread and usage of SP chewing sticks in the world [50]. Today, both the traditional SP stick and the modern toothbrush are used in conjunction around the world, most notably in Muslim countries [7,50].

In recent years, numerous studies have begun to examine the therapeutic properties of SP and its bioactive constituents in a variety of medical fields, as well as the possible roles it could play in the future as part of clinical therapies or disease prevention measures [46,51,52].

### 3.2. Plant Description and Classification

*Salvadora persica*, is plant species of the Salvadoraceae family. Its complete classification can be found in (Table 1).

SP is an upright evergreen plant with a crooked trunk that grows as a small tree or shrub. It is also known as Arak or “tooth brush tree.” It seldom grows larger than one foot in diameter and can reach a height of around three meters. The leaves are thick, small, rounded to ovate, and smell strongly of cress or mustard. The fragrant flowers are very small. The fruits, which resemble fleshy berries, are tiny and scarcely visible. They are edible both fresh and dried. The plant can live in harsh conditions and can tolerate soils ranging from intensely dry to highly saline [7]. The SP tree is widely spread in Africa, the Arabian desert, and the Indian Peninsula due to its ability to withstand a variety of conditions [7].

### 3.3. Chemical Composition of Salvadora persica

**Figure 1:**
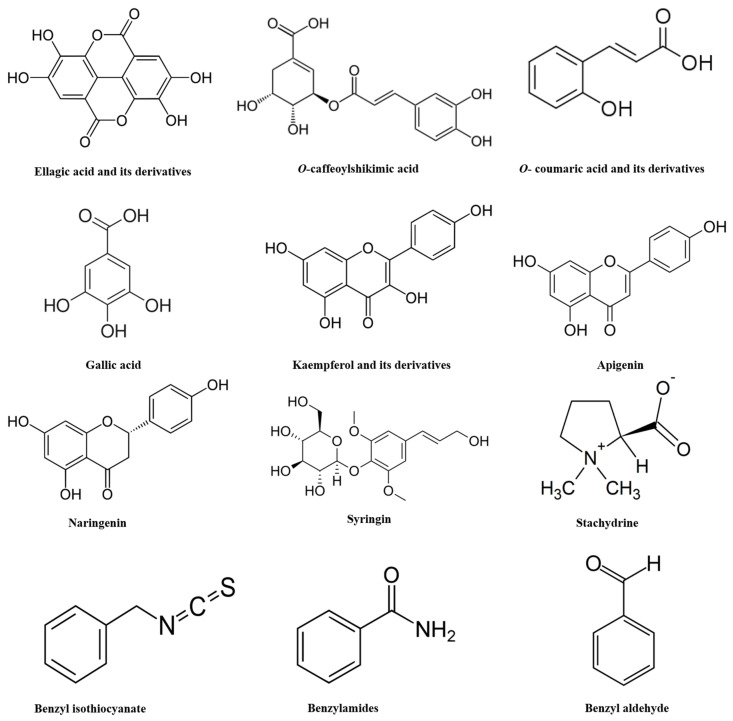
Chemical structures of important phytoconstituents present in the *Salvadora persica* extract.

Recent comprehensive examinations of SP revealed a secondary metabolite profile [53]. Its characterization revealed a total of 76 compounds which include flavonoids, phenolic acids, alkaloids, sulfur compounds, phenolic diterpenes, and fatty acids. The phenolic acid profile displayed methoxyellagic acid, di-O-methylellagic acid, acylated ellagic acid, O-caffeoylshikimic acid, O-coumaroylquinic acid, caffeoyl-O-hexoside, gallic acid, and its derivatives. SP also showed the presence of 8 flavonoid glycosides namely kaempferol aglycone (kaempferol-O-dihexoside, kaempferol-O-hexoside, and kaempferol-O-hexosyl-pentoside), isorhamnetin glycosides (isorhamnetin-O-hexoside and isorhamnetin-O-deoxyhexoide), apigenin glycosides, and naringenin-O-hexosyl-deoxyhexoside sulfate. Furthermore, SP exhibited sulfur-containing compounds as salvadoside and persicaline. Among the alkaloids detected in SP were hydroxystachydrine, hydroxylpyrrolidine, prolinebetaine, N-benzylamine, N-benzyl-2-phenylacetamide, and reticuline. Sterols and fatty acids were also identified in the extract, including stigmasten-diol, hydroxy-octadecadienoic acid, stigmasterol, β-sitosterol, palmitic acid, linoleic acid, archidic acid, and β-sitosterol-O-hexoside [53]. Investigations further observed the presence of betaines (stachydrine and 4-hydroxystachydrine), as well as 5 known (kaempferol-3,7-di-O-β-glucopyranoside, kampferol-3-O-β-glucopyranoside, quercetin-3′,7-dimethylether, isobiflorin, biflorin) and one novel cytotoxic chromen derivative known as 5-hydroxy-7-methoxy-2-methyl-4H-chromen-4-one-6-C-glucopyranoside [53]. Table 2 and Figure 1 present important phytoconstituents present in the SP extract and their potential activities.

### 3.4. Toxicological Profile of Salvadora persica

Several studies have investigated potential acute and sub-chronic toxicities of SP and its extracts on in vivo and in vitro levels (Table 3).

Safety profile of aqueous alcoholic extracts of SP roots was evaluated in vivo in a rat study model [60]. Rats were administered with up to 5 g/kg of the SP intraperitoneally and acute toxicity was checked. No visible sign of toxicity and mortality were seen till 7 days with 3 g/kg of the extract and LD_50_ was found to be 4 g/kg. Extracts of SP root were found safe for liver and kidney as depicted by biochemical and hematological parameters at a dose of 400 mg/kg body weight. However, extracts demonstrated negative effect on sexual hormones by reducing testosterone and increasing estrogen secretion in male rats whereas progesterone levels were decreased in the female rat group. In another study on human dental pulp stem cells [62] it was found that aqueous extracts of SP demonstrated a cytotoxic effect at a concentration of 5.75 mg/mL and caused significant cell proliferation at 0.08 mg/mL and 0.17 mg/mL after 48 h whereas alcoholic extracts (5.75–1.43 mg/mL) showed toxicity after 28 and 48 h. Furthermore, another in vitro investigation examined the effect of SP extract on human gingival fibroblasts [63]. Hexane extract (1 mg/mL) of SP showed cytotoxicity in 14% of the cells using lactic dehydrogenase assay while crystal violet assay showed toxicity in 12% of cells. Maximum cytotoxicity was reported in the ethyl acetate extract of SP as cell survival was only 40% and 66% when evaluated by lactate dehydrogenase and crystal violet assay respectively. In an oral acute toxicity test within an animal investigation, SP at a concentration of 300 and 500 mg/kg was found to be safe up to 5 g/kg of animal weight and reported no side effects of the drug administration [64].

Generally, it could be stated that SP extracts are mostly safe compounds which have an old history of human remedial practice with no significant safety issues. Nevertheless, to achieve the worldwide regulatory standards of drug safety and to determine the exact safe concentrations that should not be exceeded it is vital to perform more safety studies in the future to establish complete guidelines for these compounds and their application by different routes of administration in clinical therapy.

## 4. Modes of Action of *Salvadora persica* as Potential Adjuncts during Periodontal Therapy and in Periodontitis-Associated Settings

Many studies have looked at the efficacy of SP in periodontal disease-related environments, as well as its modes of action and potential advantages for periodontal care. These comprise of in vitro and in vivo studies, clinical trials (RCTs) and cross-sectional investigations. Relevant studies to this investigation’s subject are listed in Table 4. The observed therapeutic modes of action, the procedures and the results of these examinations are further described in the following section.

### 4.1. Antioxidant and Anti-Inflammatory Effects

Inflammatory reactions and the generation of reactive oxygen species (ROS) have been identified as important factors in the pathogenesis of periodontal disease [65]. In multiple studies, SP has been discoursed as an antioxidant and anti-inflammatory agent with therapeutic effects [8,9,66,67].

Previous research has shown that SP has an antioxidant effect due to its ability to scavenge different free radicals, which is attributed to the existence of powerful antioxidant enzymes and compounds such as flavonoids like luteolin, quercetin, and apigenin, p-coumaric, ferulic, sinapic, and cinnamic acid, and furan derivatives, as well as peroxidase, catalase, and polyphenoloxidase [9,66]. The antioxidant action of the bark leaves and seed cake extracts of SP by means of the ß-carotene-linoleic acid assay were examined in one of the investigations [68].

The SP seed contained two dominant tocopherols (-tocopherol and -tocopherol). Both compounds exhibited antioxidant properties similar to vitamin E, making them essential for human health [7]. Other investigations reported Δ5-avenasterol, beta-sitosterol, campesterol and stigmasterol in SP extracts and concluded that *SP* seeds have a very high oil content (41–42 g/100 g of seed) with a high ratio of saturated (SFA) to unsaturated fatty acids (UFA) (SFA/UFA 5–6 mean ratio) and an average to high oxidative stability [68]. Furan byproducts, as identified by Gas chromatography-mass spectrometry study of SP and antioxidant enzymes, peroxidase, catalase, and Polyphenol oxidase, can also have a strong antioxidant effect by removing 2,2-diphenyl-1-picrylhydrazyl radicals, (2,2′-azino-bis [3-ethylbenzo-thiazoline-6-sulfonic acid] radicals, and reducing molybdenum (VI) to molybdenum (V) [7]. This scavenging capacity of SP natural extracts has also been stated to be concentration-dependent, with improved abilities to remove H202 at higher levels of SP concentrations [66].

SP has also been confirmed experimentally to demonstrate effective anti-inflammatory outcomes supporting its antioxidant mechanisms [7,46,67,69]. It redesigns the nitric oxide synthase (NOS) isoforms and suppresses pro-inflammatory cytokines such as IL-1β, IL-6, IL-8, TNF-α and IFN [67,69,70,71], with a simultaneous release of α-Amylase enzyme promoting anti-inflammatory and anti-oxidative effects at the site of inflammation [72,73]. Similar to other herbal extracts [31,74], the capacity of SP to obstruct eicosanoid development may be correlated with the possible mechanism by which SP could employ this combined anti-inflammatory and anti-oxidant effect. Several herbal extracts have been shown to significantly inhibit lipid peroxidation and eicosanoid production, specifically thromboxane B and leukotrienes B4, via the COX and LOX molecular pathways [31]. Given the anti-inflammatory and anti-oxidant properties of SP, its extracts can have an important role in preventing the onset and progression of periodontal disease, as stated in the current study. The majority of the studies discussed in this study observed the clinical therapeutic effect of SP on gingival or periodontal inflammation and health in terms of gingival status and plaque accumulation (Table 4) [3,75,76,77,78,79,80,81,82,83,84,85,86]. In these inquiries, SP was introduced in the form of mouthwashes [75,77,78,79,81,82,83], toothpastes/dentifrices [80,85,86], chewing/brushing sticks [3,76,84,87] or periodontal films [88]. Moreover, SP was applied in several concentrations as a pure SP product [3,76,78,80,84,86,87] or in combination with other herbal elements [75,77,79,81,82,83,85]. In the current review all presented investigations reporting SP administration in different therapeutic forms could display a significant improvement of gingival inflammation and reduction of plaque accumulation (Table 4), proving a steady and successful clinical outcome of SP herbal adjunctive therapy in the treatment or prevention of inflammation and plaque as key factors of periodontal disease [31]. Furthermore, a number of studies compared the clinical periodontal-therapeutic effect of SP to Chlorhexidine as a main agent applied adjunctively in periodontal therapy [89] and observed similar or better effects of SP [82,83]. Yet, it has to be noted, that none of the presented studies investigated SP as an intrapocket medication as performed in other adjunctive herbal therapies [31] or recorded clinical outcomes as probing pocket depth or clinical attachment level as main indicators of periodontal health or regeneration on a clinical level [90]. Further clinical research will be needed in the future to determine the precise impact of SP when used in conjunction with mechanical scaling and root planning during periodontal therapy.

### 4.2. Antibacterial Effects

Periodontitis is a pathophysiological process which presents a multifactorial etiology defined by initiated inflammation of periodontal host tissues facilitated by the immune reaction and associated with dysbiotic dental plaque biofilms, subsequently damaging the tooth-supporting structures progressively and leading to the loss of periodontal attachment [18,97]. Following gingivitis caused by microbial biofilm production, dysbiotic modifications in the microbiome ecosystem occur in response to inflammatory and immune mediators, as well as tissue breakdown products, resulting in the activation of a variety of critical molecular pathways supporting periodontal degradation. Finally, host-derived proteases are activated, allowing for the damage of marginal periodontal ligament fibers, junctional epithelium apical migration, and bacterial biofilm apical spread along the root surface [18,97]. Historically, the major pathogens associated with periodontitis were thought to be species of the known “red bacterial complex” (*Treponema denticola, Porphyromonas gingivalis*, and *Tannerella forsythia*) [98]. However, this hypothesis was based on culture-based research, which did not go into great detail about the microbiological diversity in the samples [99]. Modern approaches have furthermore revealed other bacterial pathogens also closely associated with periodontitis such as the bacterial classes *Clostridia* and *Negativicutes*, *Erysipelotrichia* [100]; the genera *Fusobacterium, Prevotella* [101] and *Synergistes* [102]; as well as the species *Methanobrevibacter oralis, Methanosarcina mazeii, Methanobacterium curvum/congolense* [99,103,104], *Filifactor alocis* [100], and *Aggregatibacter actinomycetemcomitans* [105].

Numerous investigations have described the antimicrobic effect of SP on various types of bacteria [8,106,107]. SP extracts and products have been shown to have significant bactericidal activity against Gram-positive and Gram-negative bacteria, as well as suppressing bacterial biofilms, in a number of studies, reassuring the use of SP as an antimicrobial drug in a variety of diseases [8,108,109]. One of the proposed mechanisms of SP-facilitated bactericidal function was the targeting of bacterial membranes by Benzyl isothiocyanate (BITC), one of the active components of SP extracts [98]. Electron micrographs of periodontal pathogens revealed that SP extracts and BITC compounds may cause bacterial membrane protrusions similar to antibacterial peptides [98]. Through the breakdown of the outer bacterial membrane, bioactive constituents of SP will enter the bacterial cell and interact with the bacterial redox systems, impairing the microorganism’s ability to retain its membrane potential [98]. Such mechanism of BITC has also been reported for mitochondrial membranes [98]. Another proposed antibacterial mechanism for SP is the increased release of phytochemicals such as β-sitosterol, which have the potential to suppress genotoxic microbial substances deposited on the teeth [6]. Concurrently, dissolved anionic elements in the SP extract, such as chlorides, fluorides, sulphur, cyanides, and heavy metals, can degrade the bacterial cell wall, causing damage to the microbial transport system, preventing oxygen absorption, and causing high oxidative stress within bacterial cells, eventually leading to toxicity and death [6]. Furthermore, nonpolar compounds in SP, like basic and volatile oils, have been shown to be efficient in buffering the pH of saliva, resulting in reduced bacterial activity and disruption of the plaque layer and microbial biofilm [6]. Other research has found that when SP is combined with antibiotics, it has a synergistic antibacterial effect, demonstrating its function in bacterial resistance suppression [92]. This function, like that of several herbal preparations, may be related to the inhibition of microbial cell pump efflux [31,110].

**Figure 2:**
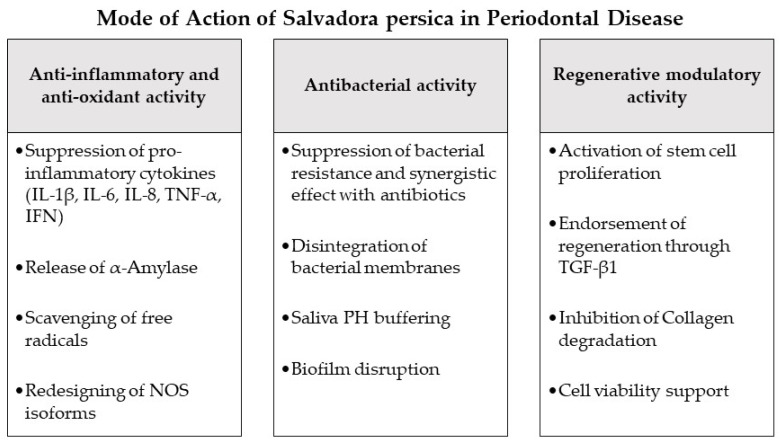
Mode of action of *Salvadora persica* as an adjunctive periodontal chemotherapeutic in periodontal disease. Abbreviations: IL-1β: interleukin 1b, IL-6: interleukin 6, IL-8: interleukin 8, TNF-*α*: tumor-necrosis-factor-*α*, IFN: interferon, NOS: nitric oxide synthase, TGF-β1: transforming growth factor-β1.

In the current investigation the reviewed studies reported in vivo and in vitro application of SP compounds on different species of periodontitis-associated bacteria [87,88,91,92,93,94,95,96]. All outcomes revealed a highly effective inhibitory and bactericidal effect of SP extract on the tested periodontal pathogens, such as *Fusobacterium nucleatum*, *Porphyromonas gingivalis*, *Aggregatibacter actinomycetemcomitans*, *Prevotella intermedia*, *Treponema denticola* and *Tannerella forsythia*. The antibacterial function was reported to be in a dose/time dependent manner with higher effects directly after SP application [87], more efficient in organic solvent extracts compared to water extracts [91,92,94] and having a coactive mechanism if used with antibiotic therapy [92]. As reported previously [52], several studies [87,88,95,96] described a higher sensitivity of SP extract against Gram negative microbes, specifically *Porphyromonas gingivalis*, as a main etiological factor of periodontal disease [111] (Figure 2 and Table 4). This may be due to the thicker peptidoglycan cell wall of the Gram positive bacteria [112], providing a higher resistance against the penetration by the SP compounds.

### 4.3. Regenerative and Stem Cell Modulation

The target of periodontal treatment is to counteract the bacterial invasion and reorganize the conformations and functions of periodontal tissues [113]. Complicated tasks arise in the attempt to regenerate the periodontal structures and reconstruct the bone-PDL-cementum complex appropriately [114]. Osteogenesis occurs slightly before the differentiation of cementum and periodontal ligament fibers. The oriented periodontal ligament fibers must then be attached to newly generated alveolar bone and cementum tissue, which is one of the most challenging processes in periodontal regeneration [115]. Numerous groups have widely discussed the significant potential of periodontal regeneration with remarkable clinical success thru mechanisms of regeneration for bone, cementum, and PDL via mobilization of endogenous stem cells from their niche areas, transplantation of exogenous stem cells targeting periodontal defects, or growth/angiogenic factor-mediated regenerative modulation [115,116,117]. Numerous mesenchymal stem cell (MSC) types persist and are responsible for tissue homeostasis, acting as a reservoir of renewable stem/progenitor cells to produce other essential cells throughout adulthood [118]. As an outcome, effective periodontal regeneration relies on the promotion of regenerative growth/angiogenic factors and the recruitment of locally-derived stem/progenitor cells, such as populations of resident periodontal or oral-tissue stem/progenitor cells to the damaged tissue site for tissue homeostasis and consequent differentiation into bone, periodontal ligament, and cementum-forming cells [115,119]. Natural herbs have been found to modulate growth/angiogenic factors, immune functions, migration, proliferation, cell fate determination, and self-renewal abilities of different types of mesenchymal stem cells in a number of studies. [31,120,121]. Among these studies, SP demonstrated a variety of effects that may influence periodontal therapy and play a role in improved regeneration and healing [51,62,67,122,123]. As elucidated in the studies [67], SP could activate transforming growth factor-β1 expression, promoting regenerative and stem cell functions, including self-renewal and differentiation, adhesion, proliferation and migration, angiogenesis, as well as production of extracellular matrix components [67,124]. SP was correspondingly able to increase MSC and fibroblast proliferation and viability [62,63], besides an inhibition of collagen degradation [123], a chief factor of periodontal destruction [125]. Other investigations reported the significantly augmented therapeutic potential and healing capacity after administration of SP extract to the damaged sites [46,51]. Such findings suggest that SP has a high regenerative accelerating potential when used in conjunction with periodontal therapy. Nonetheless, to confirm and expand our understanding of SP’s potential benefits, more in vitro and in vivo research is needed, particularly on periodontal ligament stem cells as major residents of periodontal defects (Figure 2 and Table 4).

## 5. Conclusions

Recently, there has been a rise in interest in herbal treatment as a replacement for conventional treatments or as complementary medications for a number of diseases [1,31]. Dental and oral diseases are no exception. Numerous experiments have been performed to explore the function of SP in various areas of dental science [6,7,46]. SP holds a wide range of potential therapeutic properties for numerous oral conditions. Aside from its anticariogenic effects [6,7,46], SP plays provides effective functions in the treatment or prevention of periodontal diseases, as evidenced by the current investigation. Frequent studies have shown that the susceptibility of oral and periodontal pathogens to SP extracts is equal to or better than antibiotics widely used during periodontal therapy. Furthermore, multiple in vitro and in vivo investigations have reported tangible benefits of using SP for treatment or prevention of periodontal inflammation, in addition to regular mechanical debridement, on clinical and molecular levels. These periodontal health-promoting abilities appear to be bolstered by SP rich natural extract’s superior antibacterial, antioxidant/inflammatory, and potentially regenerative mechanisms. However, more comprehensive investigations on SP-mediated periodontitis therapy are required to examine the clinical effect of SP in periodontal pockets and its precise mechanisms of action, as well as to deliver a complete overview of the outcomes of these compounds when combined with further periodontal-related treatments and medications. Overall, the reports that inspected the role of SP are still initial and may necessitate additional elaboration and expansion, mainly at RCT level for intrapocket SP application. Nonetheless, the current findings presented an astonishing potential for a future beneficial incorporation of this rich natural compound into regular periodontal therapy.

## Figures and Tables

**Table 1:** Systematic classification of *Salvadora persica*.

Kingdom	*Plantae*
Phylum	*Magnoliphyta*
Class	*Magnoliopsida*
Order	*Brassicales*
Family	*Salvadoraceae*
Genus	*Salvadora*
Species	*Persica oleoides*
Binomial classification	*Salvadora persica*

**Table 2:** Important phytoconstituents of the *Salvadora persica* extract and their reported chemical/biological activity.

Phytoconstituent	Plant Part	Reported Chemical/Biological Activity	Reference
Persicaline (Sulphur-containing imidazoline alkaloid)	Roots	Strong antioxidant properties	[54]
Salvodourea, m-anisic acid and benzyl isothiocyanate	Roots	Antiviral activity	[55]
Salvadoside, salvadoraside, syringin, and liriodendrin (lignin glycosides)	Stem	-	[56]
Essential oil components (1,8-cineole (eucalyptol), αβ-caryophellene, β-pinene, and 9-epi-(E)-caryophellene)	Stem	Anti-microbial activity	[57]
Benzylamides	Stem	Human collagen-induced platelet aggregation and antibacterial activity	[58]
Trimethylamine and salvadorine	Roots	Antibacterial, antiphlogistic and gingiva-stimulating effects	[59]
Benzaldehyde, benzyl nitrile and benzyl Isothiocyanate	Roots and twigs	Antimicrobial effects	[7]

**Table 3:** The potential *Salvadora persica* related toxicity and its reported effects.

Potential *Salvadora persica* Related Toxicity	Reported Effects	Reference
Sex hormone imbalance	Reduced testosterone and increased estrogen secretion in male rats with decreased progesterone levels in female rats	[60]
Reduced fertility	Adverse effects on male and female mice reproductive systems and fertility	[61]
Cytotoxic effects	Cell toxicity with dental pulp stem cells and gingival fibroblasts	[62,63]

**Table 4:** Main studies in relation to *Salvadora persica* of interest in periodontal health and disease.

Natural Compound	Study Type	Sample Studied, *n*	Adminitration (Dosage, Frecuency and Duration)	Main Effects	Reference
Combination of SP and CS extracts	RCT	Systemic healthy male and female aged 25–40 years without periodontitis, *n* = 14.	Combination of extracts of CS (0.25 g) + SP (7.82 mg). 15 mL rinse twice daily from baseline with follow up after 24 h.	Significant decrease in PI	[75]
SP	RCT	Systemic healthy males aged 8–10 years, *n* = 94.	Usage of SP sticks in combination with rolling brush technique 3 times per day for 3 weeks. From baseline with follow up after 3 weeks, 1- and 3 months.	Significant decrease in PI	[3]
SP	RCT	Systemic healthy participants aged 18–35 years with mild to moderate generalized marginal gingivitis, a PPD of 3 mm or less and with GI and PI higher than 1, *n* = 30	Usage of toothbrush without toothpaste versus usage of toothbrush without toothpaste in combination with SP sticks versus usage of SP sticks only. Each group performed the procedure 3 times per day. From baseline with follow up after 2, 4, 6 and 8 weeks.	Significant improvement of PI and GI after using SP adjunctive to tooth brushing	[76]
Combination of SP and AV extracts	RCT	Intubated patients aged 18–64 years hospitalized in Intensive Care Unit, *n* = 67	Mouth irrigation with herbal mouthwash (10 mg/mL SP and 940 mg/mL AV) or Chlorhexidine (0.2%) for 30 s before and after brushing the teeth every 2–3 h. From baseline with follow up after 4 days.	Significant improvement of GI after using herbal mouthwash compared to Chlorhexidine	[83]
SP	RCT	Participants aged 13–54 years with baseline PI of more than 1.	Toothbrushing with test dentifrice (SP dentrifice versus fluoride dentrifice) for 2 min twice a day. From baseline with follow up after 2 and 4 weeks.	Significant improvement of PI with SP dentrifice	[80]
Combination of extracts of SP, *Bibhitaka*, *Nagavalli*, *Gandhapura taila, Ela, Peppermint satva,* and *Yavani satva*	RCT	Systemic healthy participants aged 20–45 years with mild to moderate gingivitis and bleeding on probing, *n* = 100.	Herbal mouthwash (HiOra) along with scaling versus scaling only. 15 mL mouthwash for 30 s twice daily after food. PI and GI taken at baseline and follow at day 21.	Significant improvement of PI and GI with herbal mouthwash	[77]
Combination of extracts of SP, *Bibhitaka*, *Nagavalli*, *Gandhapura taila, Ela, Peppermint satva,* and *Yavani satva*	RCT	Systemic healthy participants aged 18–21 years, *n* = 45.	Herbal mouthwash (HiOra) 15 mL for 60 s versus Chlorhexidine (0.2%) 10 mL for 60 s versus probiotic solution 20 mL for 60 s each twice a day 30 min after toothbrushing for 14 days. PI and GI were taken at baseline with follow up at day 7 and day 14.	Significant improvement of PI and GI with herbal mouthwash	[81]
Combination of extracts of SP, Papain, Bromelain, and Neem	RCT	Systemic healthy participants aged over 18 years and undergoing fixed orthodontic treatment, *n* = 52.	Usage of toothpaste containing herbal mixture and fluoride versus standard fluoridated toothpaste only 2–3 min twice a day for 30 days. PI and GI were taken at baseline with follow up after 30 days.	Significant improvement of PI and GI with herbal toothpaste compared to the standard fluoridated toothpaste	[85]
SP	RCT	Children in chemotherapy treatment aged 6 to 12 years, *n* = 44	Additive to Chlorhexidine mouthwash SP oral drops (10 drops in 15 mL water) or normal saline (15 mL) twice a day for 2 weeks. Oral assessment guide index was recorded at baseline with follow up at day 8 and 15.	Significant improvement of plaque and gingival status with SP oral drops	[78]
Combination of extracts of SP, *Bibhitaka*, *Nagavalli*, *Gandhapura taila*, *Ela*, *Peppermint satva*, and *Yavani satva*	RCT	Dental college students, *n* = 150	Herbal mouthwash (HiOra) (5 mL) versus 0.2% Chlorhexidine (10 mL) versus saline (5 ml), each for 30 s twice daily. PI and GI recorded at baseline and follow up after 5 days.	Significant improvement of PI and GI with herbal mouthwash, similar to Chlorhexidine	[82]
SP extract and tea tree oil	RCT	Systemically healthy male and female participants aged 20–40 years, not using SP or tea tree oil- based toothpaste and with grade 2 or 3 PI on at least one of the Oral Hygiene Index teeth, *n* = 25	SP toothpaste vs. tea tree oil toothpaste. PI recorded at baseline and follow up after 24 h.	SP toothpaste showed a significant higher reduction of plaque compared to tea tree oil-based toothpaste	[86]
Combination of extracts of SP, *Bibhitaka*, *Nagavalli*, *Gandhapura taila*, *Ela, Peppermint satva*, and *Yavani satva*	RCT	Systemically healthy participants aged 20–50 years, *n* = 152	Herbal mouthwash (HiOra) (15 mL) versus 0.12% Chlorhexidine (15 mL) for 30 s twice daily. PI and GI recorded at baseline and follow up after 21 days.	Significantly higher improvement of plaque and gingival status with Chlorhexidine	[79]
SP	In Vitro	Periodontal pathogens (FN) and other oral bacteria	100 mg/mL extracts were added to cultured bacteria and incubated for 24 h at 37 °C.	Moderate to high inhibitory activity on pathogenic bacteria with no toxicity. Methanol extract was more effective compared to water extract.	[91]
SP	In Vitro	Periodontal pathogens (PG, TD, TF, AA)	SP extract (2 mg/mL) was added to cultured bacteria and incubated for 24 h. Antibiotic discs were impregnated with SP extract added to cultured bacteria and incubated for 24 h at 37 °C.	Ethanolic extract of SP showed a significant inhibitory effect on all periodontal pathogens with synergistic antibacterial effect when SP was combined with antibiotics.	[92]
Combination of extracts of SP, *Bibhitaka*, *Nagavalli*, *Gandhapura taila*, *Ela*, *Peppermint satva*, and *Yavani satva*	In Vitro and Ex Vivo	Periodontal pathogens (PG, FN, AA) and oral bacteria in supragingival plaque samples of male and female participants aged over 18 years and periodontally healthy	In Vitro: Test mouthwash was added in different solutions (20–200 μg/mL) to cultured bacteria and incubated as appropriate for the species. Ex Vivo: Test mouthwash was added to bacteria cultured from supra gingival plaque samples with incubation for 5–7 days	Significant inhibitory effect of the herbal mouthwash on the tested bacteria	[93]
SP	In Vitro	Periodontal pathogens (PTI) and other oral bacteria	Alcoholic and water extracts of SP (200 μg/mL and 400 μg/mL) were incubated with cultivated samples for 24 h at 37 °C. The inhibitory zone was measured after 24 h.	Alcoholic extract of SP showed antimicrobial effect against all tested microbial pathogens.	[94]
Combination of SP, mint and yarrow extracts	In Vitro	Periodontal pathogens (PG, AA) isolated from 50 patients with moderate to severe periodontitis	Herbal solution (6%) versus sterile distilled water versus chlorhexidine were incubated with the cultured bacteria for 48 h at 37 °C. After 48 h the zone of inhibition was measured.	Herbal solution showed significant inhibitory effect against PG with weaker effect against AA	[95]
SP	In Vivo/In Vitro	Adults with good oral health, *n* = 12. Antibacterial effect of SP essential oil on oral bacteria and periodontal pathogens (PG, AA)	Usage of fresh cut SP root versus one time-, two time- and four time used twig. Saliva sample taken before and after brushing with the SP sticks and follow up 5-, 10- and 30 min after brushing.	Highest concentration of active compounds was detected in saliva immediately after brushing with fresh SP. Bacteria growth was inhibited by SP with PG being the most sensitive.	[87]
SP	In Vitro	Periodontal pathogens (PG) and herpes simplex virus-1	SP films (100 µg per 2 cm^2^) formulated and the SP inhibitory effect tested on the cultured microorganisms	Significant inhibitory effect of the SP films against PG and the herpes simplex virus-1	[88]
SP	In Vitro	Periodontal pathogens (PG, AA) and other oral bacteria	Essential oil of SP in concentration 1%, 0.1%, 0.05%, 0.02%, 0.01%, 0.001% was incubated with cultured test bacteria for 90 min at 37 °C. Medium-pressure liquid chromatography, Thin-layer chromatography, Gas chromatography-mass spectrometry and Transmission electron microscopy were performed	SP extract and its active constituent benzyl isothiocyanate exhibited rapid and strong bactericidal effects against all Gram-negative bacteria.	[96]
SP	Cross-Sectional	287 male school children aged 12–15 years.	Participants were assigned in group I: SP stick users, group II: toothpaste/toothbrush users and group III: SP stick and toothbrush users. All individuals were interviewed regarding their oral hygiene habits. Oral Hygiene, PI and GI were recorded.	Statistically significant differences of GI was observed among SP and toothbrush & toothpaste users as SP users had lower GI scores. PI was lowest among combined users of toothbrush and miswak.	[84]

Abbreviations: PI: Plaque index; GI: Gingival index; FN: *Fusobacterium nucleatum*; PG: *Porphyromonas gingivalis*; AA: *Aggregatibacter actinomycetemcomitans*; PTI: *Prevotella intermedia*; TD: *Treponema denticola*; TF: *Tannerella forsythia*; SP: *Salvadora persica*; CS: *Camellia sinensis.*
